# Supplementary material for: MetaRibo-Seq measures translation in microbiomes
Source: Nat Commun. 2020 Jun 29;11:3268. doi: 10.1038/s41467-020-17081-z (PMC7324362; doi:10.1038/s41467-020-17081-z)
Supplement: Supplementary file 10 — Supplementary Data 7 [file 41467_2020_17081_MOESM10_ESM.zip › File2/Confidence_High_Taxonomy/260076_out.krona.html]

Javascript must be enabled to view this page.

members
magnitude
magnitudeUnassigned
count
unassigned
taxon
rank

260076\_out

54

1

SRS104975\_contig\_number\_115
superkingdom
2
53

45
1239
phylum

1

SRS147557\_contig\_number\_12100
species
1263002

44
186801
class

44

SRS019161\_contig\_number\_26666
1
186802
order

1392389
genus
1

1297617
species

SRS1055083\_contig\_number\_10578
1

216572
family
8

3

SRS011134\_contig\_number\_contig-100\_2260.272074SRS063518\_contig\_number\_contig-100\_3011.196578SRS078419\_contig\_number\_contig-100\_2792.193110
genus
459786
8

5

SRS012849\_contig\_number\_6966SRS021948\_contig\_number\_4205SRS104400\_contig\_number\_13870SRS893383\_contig\_number\_371SRS971276\_contig\_number\_3667
species
1945593

species
1898207
2

SRS020394\_contig\_number\_2372SRS047433\_contig\_number\_586

6
family
541000

1
1263
genus


SRS015782\_contig\_number\_39288
1
41978
species

genus
244127
4

4

SRS016203\_contig\_number\_contig-100\_4427.64767SRS1041032\_contig\_number\_9406SRS1041141\_contig\_number\_12131SRS148196\_contig\_number\_21782
species
1262703


SRS1041145\_contig\_number\_25344
1
1952440
species

5

SRS017821\_contig\_number\_18653SRS045004\_contig\_number\_10964SRS045645\_contig\_number\_30824SRS1041133\_contig\_number\_13364SRS1041136\_contig\_number\_8131
species
1897049

family
424536
1

1
270497
genus


SRS017916\_contig\_number\_9437
1
1946238
species


SRS150029\_contig\_number\_contig-100\_3898.95545
1
172733
species

16

SRS015663\_contig\_number\_7786SRS018836\_contig\_number\_14784SRS022609\_contig\_number\_32147SRS048164\_contig\_number\_18526SRS049995\_contig\_number\_12818SRS052697\_contig\_number\_23611SRS058070\_contig\_number\_29462SRS064757\_contig\_number\_1189SRS075773\_contig\_number\_8931SRS078176\_contig\_number\_22585SRS101376\_contig\_number\_1357SRS143895\_contig\_number\_6259SRS144537\_contig\_number\_contig-100\_345.261069SRS147271\_contig\_number\_26286SRS147346\_contig\_number\_25718SRS149784\_contig\_number\_3873
species
1897045

3
186803
family

species
1898203
1

SRS051031\_contig\_number\_13090


SRS018541\_contig\_number\_9123SRS1041092\_contig\_number\_8525
2
39491
species

1224
phylum
6

28216
class
5

80840
order
5

family
506
1

genus
222
1

1

SRS1041033\_contig\_number\_contig-100\_10929.91004
species
1806492

119060
family
4

4
genus
106589

species
574750
4

SRS054059\_contig\_number\_3968SRS063813\_contig\_number\_contig-100\_6471.6472SRS1041140\_contig\_number\_contig-100\_31206.67093SRS142503\_contig\_number\_54067

subphylum
68525
1


SRS075341\_contig\_number\_contig-100\_350.109414
1
28221
class

201174
phylum
1

class
1760
1

85011
order
1

2062
family
1

genus
1883
1

SRS019445\_contig\_number\_1880

1

SRS1055038\_contig\_number\_contig-100\_1565.1566
